# Community Interagency Connections for Immigrant Worker Health Interventions, King County, Washington State, 2012–2013

**DOI:** 10.5888/pcd13.160013

**Published:** 2016-06-02

**Authors:** Jenny Hsin-Chin Tsai, Miruna Petrescu-Prahova

**Affiliations:** Author Affiliations: Miruna Petrescu-Prahova, University of Washington, School of Public Health, Seattle, Washington.

## Abstract

**Introduction:**

Cross-sector community partnerships are a potentially powerful strategy to address population health problems, including health disparities. US immigrants — commonly employed in low-wage jobs that pose high risks to their health — experience such disparities because of hazardous exposures in the workplace. Hazardous exposures contribute to chronic health problems and complicate disease management. Moreover, prevention strategies such as worksite wellness programs are not effective for low-wage immigrant groups. The purpose of this article was to describe an innovative application of social network analysis to characterize interagency connections and knowledge needed to design and deliver a comprehensive community-based chronic disease prevention program for immigrant workers.

**Methods:**

Using iterative sample expansion, we identified 42 agencies representing diverse community sectors (service agencies, faith-based organizations, unions, nonprofits, government agencies) pertinent to the health of Chinese immigrant workers. To capture data on shared information, resources, and services as well as organizational characteristics, we jointly interviewed 2 representatives from each agency. We used social network analysis to describe interagency network structure and the positions of agencies within the networks.

**Results:**

Agency interconnections were established primarily for information sharing. In the overall interagency network, a few service-oriented agencies held central or gatekeeper positions. Strong interconnectedness occurred predominately across service, public, and nonprofit sectors. The Chinese and Pan-Asian service sectors showed the strongest interconnectedness.

**Conclusion:**

Network analysis yields critical understanding of community structural links and assets needed to inform decisions about actual and potential community collaborations. Alternative intervention strategies may be needed to address health disparities among immigrant workers.

## Introduction

Health concerns in the United States are focused on disparities in access to health care and health outcomes. Of particular concern are occupational health disparities associated with race/ethnicity and immigrant status (1). Immigrant racial/ethnic minority populations, compared with their native-born counterparts, have a disproportionate share of worksite hazard exposures and associated health problems (2). Many workplace hazard exposures contribute to chronic health problems, such as musculoskeletal disorders, mental disorders, and cancer (3), and complicate the management of existing chronic illnesses (4,5). Worksite prevention and wellness programs — promoted by the US Affordable Care Act (6) — are not effective for immigrant workers employed in low-wage jobs (7,8). This deficiency is linked to the lack of effective methods to engage individuals in this vulnerable, hard-to-reach population.

A promising approach to advance prevention efforts for immigrant worker health is cross-sector community collaboration that involves both cultural and linguistic community resources (9–12). This approach focuses on community agency networks to strengthen community infrastructure and capacity and provides a cost-effective and sustainable strategy to improve population health (13,14). Effectively engaging interagency networks for health promotion and prevention, however, requires comprehensive understanding of the structural linkages among community resources. Extant research provides little guidance for strategically identifying and engaging immigrant community institutions for promoting worker health and reducing occupational health disparities.

The purpose of this article was to describe an innovative application of social network analysis to identify interagency connections needed to design and deliver comprehensive community-based chronic disease prevention programs for Chinese immigrant workers. 

## Methods

This study, conducted from 2012 to 2013, used a cross-sectional, descriptive design to characterize community interagency networks that support Chinese immigrant worker health. Institutional review board approval was obtained from our affiliated university before recruitment. Participating agencies were located or had an office in King County — the county with the highest Chinese population density (3.8%) in Washington State (15). Agencies corresponded to diverse community sectors: Chinese service agencies, Chinese faith-based organizations (FBOs) (churches and temples), Pan-Asian service agencies, labor unions, pan-ethnic nonprofit agencies, and public (government) agencies. Historically, Chinese service and faith-based organizations have provided formal and informal support to Chinese immigrants (16,17). In comparison, other agencies and organizations have typically served a diverse range of clientele, including Chinese immigrants. In this study, identified agencies served Chinese immigrants or had a service mission and scope relevant to worker health (eg, job training, advocacy, occupational health training). The initial sampling roster included 40 agencies drawn from available community directories. An expanded roster (18,19) was subsequently generated by asking interviewees to nominate other community agencies important to Chinese immigrant worker health. Nominated agencies identified by at least 2 respondent agencies were included in the expanded roster. The use of at least 2 separate nominations facilitated inclusion of relevant network agencies not on the initial roster and exclusion of those not part of the network (19). The final roster of 42 agencies included 5 Chinese service agencies, 11 Chinese FBOs, 6 Pan-Asian service agencies, 3 unions, 12 pan-ethnic nonprofits, and 5 public agencies.

Prospective interviewees were identified by agency administrative contact followed by interview invitations extended to appropriate agency personnel. First, we contacted agency directors to obtain their agreement to participate. After receiving a letter of agreement from each agency, we worked with each director to identify an administrator and a service staff member meeting study criteria, which included being 1) proficient in English or Chinese, 2) in an agency position for at least 12 months to ensure familiarity, and 3) knowledgeable about the range of agency activities. We anticipated that administrators would have more knowledge than would service staff of administrative linkages such as joint programs and service contracts, and that service staff would be more aware than would administrative staff of service delivery linkages, such as referrals (20). When directors recommended 2 or more prospective interviewees, either administrative workers or staff, we randomized the order for contact. To assure human subjects’ protection and data validity, agency directors were informed that prospective interviewees’ decisions to participate or not participate would be kept confidential.

Data collection involved joint interviews with 2 staff members from each agency. Trained bilingual (English and Chinese) research interviewers administered structured interviews to collect data on organization characteristics (eg, mission, size, bilingual capacity, programs, populations served) and organizational network relationships (20,21). Network questions assessed 6 types of cross-agency relationships: information sharing, resource sharing, referrals, joint programs, joint political actions, and service contracts. Interviewees identified their agency’s links (0 = no link, 1 = linked) with all other agencies listed with respect to the 6 types of relationships. Information and resource sharing were considered “directed” relationships because agencies were asked to report only about *sending* information (or resources) *to* other agencies (eg, “Does your agency/organization share information relevant to Chinese immigrant worker health, employment-related and other assistance, or advocacy at least once a month with the agency/organization listed during the past 12 months?”). Questions about the other 4 types of relationships (referrals, joint programs, joint political actions, and service contracts) were “undirected.” That is, they inquired about *mutual* agency interactions (eg, “Does your agency/organization send or receive Chinese immigrants for worker health-related issues at least once a month to or from the agency listed during the past 12 months?”). Using responses about the 6 relationship types, we generated 6 interagency networks and calculated network properties (Table 1). Density, centralization, and link strength are network-level properties describing network structures; centrality is a node-level (agency level) property characterizing each agency’s positions in a given network (22,23). The interview was pilot tested with 1 Chinese FBO and 1 community-based organization (CBO), which were not on the sample roster, and we found that minor wording refinement was needed.

**Table 1:** Definitions and Measures for Network-Level and Node-Level Properties, Community Interagency Connections for Immigrant Worker Health Interventions, King County, Washington State, 2012–2013

Network Properties	Definition	Measures
**Network-level properties**		
Density	Proportion of possible links present in network	• Count links; divide by number of possible links • Values range from 0 (no links) to 1 (all entities linked with one another)
Degree centralization	Extent to which network is dominated by one or a few high-degree agencies (those with the greatest number of links)	• Calculate from degree-centrality scores • Values range from 0 (a decentralized network; all agencies had the same number of links) to 1 (a completely centralized network with one central agency)
Betweenness centralization	Extent to which network is dominated by one or a few high-betweenness agencies	• Calculate from betweenness-centrality scores • Values range from 0 (all agencies were directly connected to other agencies) to 1 (links were between one agency and all other agencies)
Link strength (multiplexity)	Extent to which network agencies are linked through one or multiple pathways	• Sum total responses to all survey questions on relationships • Values range from 0 (not linked) to 6 (linked for all 6 types of relationships)
**Node-level properties**		
Degree centrality	Extent to which an agency is connected to other network agencies	• Sum direct links to any one agency in the network
Betweenness centrality	Extent to which an agency is in a gatekeeper position, bridging pairs of agencies and controlling information exchange or resource flows by virtue of location within the network paths	• Count number of paths from one agency (A) to the other agency (B) that pass through a 3rd agency, divided by number of paths from Agency A to Agency B

We used descriptive statistics to evaluate organizational characteristics and social network analysis methods to depict the patterns of relationships among agencies and the roles agencies played in community interagency networks. Using UCINET 6 (24), we computed density, centralization, and centrality and created network sociograms for each relationship. High density scores (range, 0–1) indicate a high proportion of possible links present in the network, which suggests a cohesive network (Table 1). High degree centralization scores (range, 0–1) indicate a centralized network dominated by a small number of agencies, whereas high betweenness centralization suggests a network with a small number of agencies acting as gatekeepers or bridges that may control network information and resource flow. A high degree centrality score means that an agency has many direct links with other agencies on the roster; a high betweenness centrality score indicates an agency’s potential to serve as a network gatekeeper or bridge connecting pairs of agencies.

For undirected relationships, we analyzed the data in 2 ways: using confirmed and unconfirmed links. Confirmed links (both agencies in a pair reported the link) offered a more dependable indicator of actual interactions. Unconfirmed links (only 1 agency in a pair reported the link) provided information useful to understand agencies’ impression of their involvement with other agencies. The discrepancies between confirmed and unconfirmed networks provided information on potential network connections for future community infrastructural building (25).

For cross-sector network relationships (eg, between the Chinese FBO sector and the public agency sector), we examined the strength of links (or multiplexity) between sectors and developed sociograms. We first created an aggregate directed network comprising the information and resource sharing networks and an aggregate undirected network comprising the remaining 4 networks. Within these 2 aggregate networks, link strength was equal to the number of overall links between the 2 agencies. For instance, if agencies A and B were involved in both referrals and joint programs, the link value in the aggregate undirected network was 2; if agencies A and B were also involved in service contracts, the link value was 3. A high value means a high level of involvement between agencies through different relationships. Because the sample size differed among sectors, we also calculated density (ie, proportion of possible links observed in the network) as the average cross-sector link strength for each aggregate network.

## Results

Thirty-six of the 42 agencies on the final roster participated, yielding an agency acceptance rate of 86%. Twenty-eight respondent agencies had 250 or fewer paid staff members; 6 agencies had no paid staff. All agencies were supported by multiple funding sources; individual or community donation was the most commonly used mechanism to sustain operation (n = 30, 83%). Twenty-three agencies had staff members dedicated to working with immigrants who used their services during the past 12 months.

### Overall interagency network structures and agency positions

Table 2 describes descriptive results of the 2 directed networks and Table 3 describes results of the 4 undirected interagency networks. Density analysis revealed that agencies were more likely to share information than resources (Table 2). For an ad hoc analysis we computed network reciprocity, a measure relevant to directed networks only, to evaluate the degree to which the proportion of links among agencies were reciprocated or mutual. The reciprocity scores reflecting mutual exchanges among agencies were similar: 43% of the links show mutual sharing of information and 41% show sharing resources. Centralization scores revealed that information sharing was concentrated on a small number of agencies more than resources sharing.

**Table 2:** Network Analyses of Directed Networks^a^: Information and Resource Sharing (N = 42), Community Interagency Connections for Immigrant Worker Health Interventions, King County, Washington State, 2012–2013

Relationship	Density	Centralization (range 0–1)			Centrality	
		Outdegree^b^	Indegree^c^	Betweenness	Degree^d^	Betweenness^d^
Information sharing	0.16	0.76	0.26	0.17	PAN 05 PUB 01 SER 04 PUB 03 NON 03	PAN 05 NON 05 SER 01 SER 04 PUB 03
Resource sharing	0.10	0.52	0.20	0.11	PAN 05 NON 06 PUB 03 NON 05 NON 03	PAN 05 PUB 03 SER 04 NON 05 NON 03

Abbreviations: NON, nonprofit; PAN, Pan-Asian agency; PUB, public agency; SER, Chinese service agency.

a Reflect directional relationships sending information or resources from one agency to another.

b Calculated on the basis of links sent from the agency that answered the question.

c Calculated on the basis of links received from other agencies that answered the question.

d Numbers after the abbreviations of community sector types refer to agency code numbers.

**Table 3:** Undirected Networks^a^: Referrals, Joint Programs, Joint Political Actions, and Service Contracts of 42 Agencies, Community Interagency Connections for Immigrant Worker Health Interventions, King County, Washington State, 2012–2013

Relationships	Density	Centralization		Centrality	
		Degree	Betweenness	Degree^b^	Betweenness^b^
**Unconfirmed^c^**					
Referrals	0.15	0.58	0.37	PAN 05 SER 04 NON 05 PUB 03 SER 01	PAN 05 SER 04 NON 02 NON 05 PAN 04
Joint programs	0.09	0.42	0.30	PAN 05 NON 05 NON 10 PUB 03 PUB 04	PAN 05 NON 05 FBO-C 03 PUB 04 NON 10
Joint political actions	0.06	0.32	0.16	PAN 05 NON 05 NON 09 PAN 04 SER 04	PAN 05 PAN 04 NON 09 NON 05 SER 04
Service contracts	0.06	0.30	0.11	NON 03 PAN 05 PUB 03 NON 05 NON 01	NON 03 PAN 05 NON 05 PUB 04 PBU 03
**Confirmed^d^**					
Referrals	0.04	0.26	0.06	PAN 05 SER 01 NON 09 SER 04 NON 08	PAN 05 SER 01 PAN 06 NON 09 SER 04
Joint programs	0.02	0.14	0.04	PAN 05 NON 05 NON 09 FBO-C 02 NON 01	PAN 05 SER 04 NON 01 NON 05 NON 08
Joint political actions^e^	0.01	0.17	0.03	NON 05 PAN 05 NON 09 NON 08 UNI 03	NON 05 PAN 05 SER 04 – –
Service contracts	0.02	0.16	0.05	PAN 05 NON 03 NON 08 NON 05 PUB 04	PAN 05 NON 03 NON 08 NON 05 PUB 04

Abbreviations: FBO, Chinese faith-based organization; NON, nonprofit; PAN, Pan-Asian agency; PUB, public agency; SER, Chinese service agency; UNI, union.

a Reflect mutual agency interactions.

b Numbers after the abbreviations of community sector types refer to agency code numbers.

c Links coded as present if only one of the agencies in the pair reported the link.

d Links coded as present when both agencies in the pair reported the link.

e Only 3 agencies had a betweenness centrality score greater than 0.

For networks based on undirected relationships (Table 3), density scores showed that agencies were approximately 2 to 3 times as likely to be involved in making or receiving referrals as in joint programs, joint political actions, or service contracts. This pattern was also evident in the centralization results (Table 3), where the network based on referrals was the most centralized. As would be expected, calculating scores based only on confirmed links led to lower density and centralization scores across the undirected networks compared with results obtained when only unconfirmed links were used. Nonetheless, when scores were based only on confirmed links, the referrals network remained the most dense and centralized structure among the 4 undirected networks.

Centrality analysis (Tables 2 and 3) showed that a few agencies held central or gatekeeper positions in the networks and had the highest levels of interaction with other agencies. Across all networks, one Pan-Asian agency (PAN 05) dominated the central *and* gatekeeper positions; this pattern held across networks except for service contracts network, where one nonprofit (NON 03) dominated. Other agencies among the top 5 *across* all networks included one Chinese service agency (SER 04), one nonprofit (NON 05), and 2 public agencies (PUB 03, PUB 04). One Chinese FBO had a high betweenness centrality score in the unconfirmed joint programs network; unions did not occupy central or gatekeeper positions in any of the unconfirmed networks. The confirmed networks showed a similar pattern in numbers of agencies with high centrality scores but with minor changes in the top 5 agencies.

### Cross-sector interagency network structures

**Figure 1:**
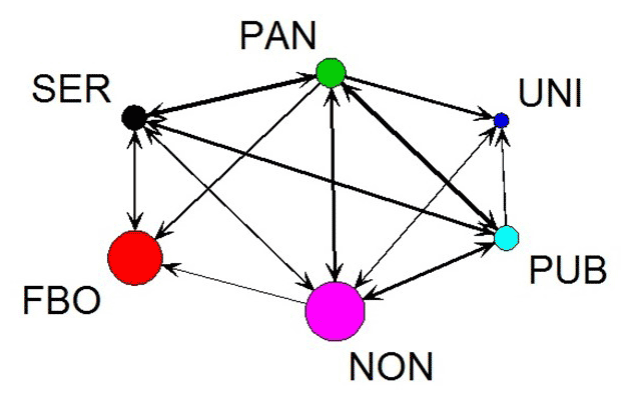
The aggregate directed network comprising the information and resource sharing networks. Colored circles (or nodes) correspond to agency sectors. Circle size is proportional to the number of agencies in each sector; line width is proportional to the density of links between sectors. Arrow heads indicate the direction of the interaction. Abbreviations: FBO, faith-based organization; NON, nonprofit; PAN, Pan-Asian agency; PUB, public agency; SER, Chinese service agency; UNI, union. **Table:** 

**Figure 2:**
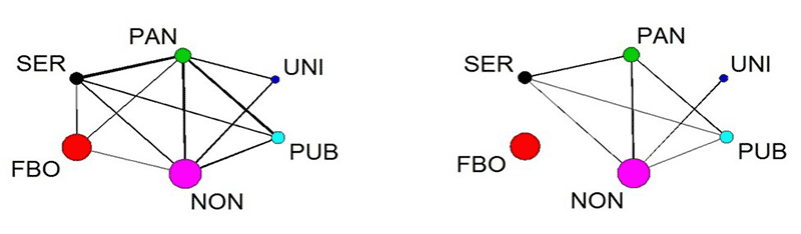
The aggregate undirected network comprising the referrals, joint programs, joint political actions, and service contracts networks. Colored circles (or nodes) correspond to agency sectors. Circle size is proportional to the number of agencies in each sector; line width is proportional to the density of links between sectors. Abbreviations: FBO, faith-based organization; NON, nonprofit; PAN, Pan-Asian agency; PUB, public agency; SER, Chinese service agency; UNI, union. **Table:** 

Cross-sector density scores ≥ 0.50 indicate that at least half of the possible links among the agencies in paired sectors were observed in the network. Multiplexity analysis of the aggregate directed network (Figure 1) revealed high density levels for information and resource sharing between the Chinese service agency sector and the Pan-Asian agency sector (Chinese to Pan-Asian service = 0.63; Pan-Asian to Chinese service = 0.60); between the public agency sector and Chinese service sector (public to Chinese service = 0.56; Chinese to public service = 0.32); between the public agency sector and the Pan-Asian agency sector (public to Pan-Asian service = 0.67; Pan-Asian to public service = 0.70); and between the public agency sector and nonprofit sector (public to nonprofit = 0.50; nonprofit to public = 0.33). For aggregate undirected networks (Figure 2), high levels of interactions across referrals, joint programs, joint political actions, and service contracts occurred between the Pan-Asian agency sector and the Chinese service sector (unconfirmed = 0.87, confirmed = 0.40), public agency sector (unconfirmed = 0.77, confirmed = 0.23), and nonprofit sector (unconfirmed = 0.71, confirmed = 0.31). Chinese FBO and union sectors had relatively few cross-sector relationships: FBOs did not interact with unions or public agencies, and unions did not interact with Chinese service agencies.

Agency	SER (n = 5)	FBO (n = 11)	PAN (n = 6)	UNI (n = 3)	PUB (n = 5)	NON (n = 12)
SER	0.70	0.31	0.63	0.00	0.32	0.20
FBO	0.13	0.05	0.00	0.00	0.00	0.00
PAN	0.60	0.17	0.70	0.33	0.70	0.42
UNI	0.00	0.00	0.00	0.83	0.00	0.14
PUB	0.56	0.00	0.67	0.07	0.85	0.50
NON	0.20	0.04	0.44	0.14	0.33	0.74

Agency	SER (n = 5)	FBO (n = 11)	PAN (n = 6)	UNI (n = 3)	PUB (n = 5)	NON (n = 12)
**Unconfirmed network**						
**SER**	0.70	—	—	—	—	—
**FBO**	0.20	0.13	—	—	—	—
**PAN**	0.87	0.18	1.40	—	—	—
**UNI**	0.00	0.00	0.28	1.33	—	—
**PUB**	0.28	0.00	0.77	0.00	0.50	—
**NON**	0.38	0.02	0.71	0.39	0.44	1.00
**Confirmed network**						
**SER**	0.30	—	—	—	—	—
**FBO**	0.00	0.04	—	—	—	—
**PAN**	0.40	0.00	0.13	—	—	—
**UNI**	0.00	0.00	0.00	0.00	—	—
**PUB**	0.04	0.00	0.23	0.00	0.00	—
**NON**	0.05	0.00	0.31	0.08	0.03	0.32

## Discussion

Examining 6 types of relationships revealed that interagency interactions existed primarily through sharing of information. A few public agencies, service agencies, and nonprofits were the main “senders” of information. Similarly, a few agencies accounted for the most interactions with other agencies, which flagged their prominent positions and roles in serving Chinese immigrants in the area. At the sector level, strong interconnectedness occurred across service agency, public agency, and nonprofit sectors; the Chinese and Pan-Asian service agency sectors showed the strongest interconnectedness. Despite the functions of Chinese FBOs for Chinese immigrants’ lives and the functions of unions for worker health and job-related issues, these 2 sectors had few interactions with other community sectors.

Our study findings are relevant to community-based approaches for immigrant worker health, including work- and nonwork-related health problems. Interagency networks are effective mechanisms for intervention delivery to reach a broader range of community groups, for program maintenance and optimal use of resources, and for capacity building to promote healthy populations and communities (13,21,26). For many nonoccupational health problems in marginalized populations, community-based collaborative approaches are being used to address individual- and system-level health issues (11,27). Occupational health researchers, on the other hand, have collaborated with unions, CBOs, and occasionally, other community partners (eg, Spanish-language radio station, churches) to engage worker populations that are otherwise difficult to reach (9,28). Rarely, however, do such efforts start with understanding how existing interagency networks in the target communities benefit from existing networks and infrastructures and optimize the diffusion of innovation, efficiency of program delivery, and resources use. We found that private and public agencies interact with one another in multiple forms (information, resources, referrals, programs, political actions, and contracts) to support immigrant workers’ health and employment-related needs. Consistent with network research on coalitions (25), network cohesion is influenced by the reasons for interconnections. Information sharing is the most common reason for collaboration, partly because it requires few agency resources. Importantly, information sharing is a step toward closer working relationships for related resource sharing, making or receiving referrals, providing joint programs, and building community capacity (25). Our research provides a knowledge base for expanding community collaboration beyond information sharing, to include more intensive activities such as jointly offering programs and collaborating on political actions for community-based interventions.

The findings identify links within the community structure that can support immigrant worker health. Interconnectedness was greater among service-oriented sectors than connections between and among Chinese FBOs or unions. This might be due to the similarity of these agencies’ functions and focuses, reflecting a “homophilous tendency” or institutional preference to collaborate with similar institutions, reported in other network studies (29). We also examined unconfirmed and confirmed networks because differences in their structures revealed agency connections, sometimes unrecognized, that could be developed to strengthen community relationships (25). Existing and stable interconnectedness among service-oriented sectors is an untapped community asset.

Finally, partnering with co-ethnic agencies or groups is vital to establish credibility, to build trust, and to serve as a point of entry to an immigrant community to address immigrant workers’ experience with occupational health disparities (11). Our study findings suggest that co-ethnic service agencies such as Chinese and Pan-Asian service agencies are well-positioned to serve as strategic partners needed to implement prevention efforts. Chinese FBOs, however, showed weak connections. Chin and colleagues (16) suggest that the organization’s understanding of its roles in the community influences an FBO’s decisions to become involved in public health initiatives. FBOs that are more progressive in their view of social justice are more likely to collaborate on social or stigmatized issues such as workers’ rights and human immunodeficiency syndrome/AIDS programs (16). More research is needed to understand how to engage a broad range of FBOs in promoting immigrant worker health.

The analysis relied primarily on self-reported data; however, we used several mechanisms to minimize potential biases. We jointly interviewed one administrator and one staff person from each agency (20,30) and encouraged interviewees to consult with other agency personnel if clarification was needed. We examined both unconfirmed and confirmed links (30) and found consistent patterns, though with lower scores, as expected, for confirmed links.

Using a novel methodological approach, this research provides insights into community networks and their assets needed to forward cross-sector collaboration directed toward immigrant worker health. Evidence of strong vs weak interconnectedness and central and gatekeeper agencies is useful to determine stable interagency relationships within the community. This information could be used to strengthen the health promotion infrastructure, to diffuse innovative interventions, and to identify agencies that could effectively serve as partners to achieve program goals (21). One implication for intervention is to partner strategically with agencies in central or gatekeeper positions, specifically in the information sharing network. These key partners can help disseminate educational materials about worker health to immigrant clients, which will diffuse information through interagency connections. Another intervention strategy would be to increase programmatic-related linkages among the service-oriented sectors. Research replication with similar community sectors and other ethnic communities will deepen our understanding of actual and potential FBO and union connections with private and public service agency sectors. Such knowledge is needed to formulate strategies for strengthening network connections with FBOs, unions, or both, to address immigrant worker health.

Community agency networks are assets for improving population health. To our knowledge, this is the first occupational health study examining interagency networks across community sectors pertinent to immigrant worker health. Social network analysis is an analytical approach that can be valuable in evaluating both network structural change (25) in community partnerships and capacity development in response to community-based interventions designed to improve immigrant worker health.
